# Determinants of place of death: a population-based retrospective cohort study

**DOI:** 10.1186/1472-684X-12-19

**Published:** 2013-05-01

**Authors:** Jyothi Jayaraman, KS Joseph

**Affiliations:** 1 Division of Palliative Care, Department of Family Medicine, University of British Columbia, Vancouver, BC, Canada; 2 School of Population and Public Health and the Department of Obstetrics and Gynaecology, University of British Columbia, Vancouver, BC, Canada; 3 Vancouver General Hospital, 899 12th Avenue W, Vancouver, BC V5Z 1M9, Canada

**Keywords:** Palliative care, Death, Home, Immigrants, Canada

## Abstract

**Background:**

As Canada’s population ages, the location of end of life care (whether at home, extended care facility or hospital) may change depending on the location of death. We carried out a study to identify determinants of the place of death.

**Methods:**

Data on deaths in British Columbia between 2004 and 2008 were obtained from the Vital Statistics Agency. Place of death was categorized into home, extended care facility, hospital or other. Logistic regression analyses were used to estimate the effects of age, sex, marital status, residence, place of birth and cause of death on place of death using adjusted odds ratios and 95% confidence intervals (95% CI).

**Results:**

Of the 153,111 deaths in the study, 16.5% occurred at home, 29.0% in extended care, 51.0% in hospital and 3.5% occurred elsewhere. Male deaths were less likely to occur in extended care as compared with female deaths (odds ratio 0.73, 95% CI 0.71–0.75). Age (odds ratio 3.31, 95% CI 3.19–3.45 for those for ≥90 vs 70–79 years), marital status (odds ratio 1.42, 95% CI 1.38–1.47 widowed vs married), residence (odds ratio 0.80, 95% CI 0.76–0.83 rural vs Vancouver), place of birth (odds ratio 0.80, 95% CI 0.75–0.86 China vs Canada) and cause of death (odds ratio 3.91, 95% CI 3.69–4.13 dementia vs cancer) were also associated with death in extended care.

**Conclusions:**

Information on determinants of place of death can inform public health policy regarding care at the end of life and make resource allocation more efficient.

## Background

In 1950, approximately 50% of deaths in Canada occurred in hospital. This rate increased steadily and peaked in 1994 when about 81% of Canadian deaths occurred in hospital [1]. There has been a steady and sharp decline in hospital deaths since and approximately 61% of deaths in Canada occurred in hospital in 2004 [2]. Meanwhile, the percentage of deaths occurring at home has remained relatively constant in recent years, while the proportion of deaths in extended care facilities has increased substantially over the last decade. In 2009, about 30,000 people died in British Columbia, with the majority of deaths occurring in hospital (about 54%), while the rest occurred at home (16%), in extended care facilities (27%) and elsewhere [3].

Rates of death in hospital, at home and in extended care vary across Canada, with place of death influenced by demographic and sociologic factors such as age, sex and urban versus rural residence [4]. This issue has implications for public health policy given recent changes in the Canadian population including its ageing and changing ethnic composition. A better understanding of where specific subgroups of people die has implications for resource allocation for end of life care services in hospitals, in long term care facilities and in the community.

A recent study examined the effect of some of these determinants in Western Canada [5]. However, variations in recording of place of death among the different provinces did not permit a sufficiently detailed exploration of the independent determinants associated with place of death. We therefore carried out a study to quantify the independent contributions of various determinants of place of death in British Columbia. Determinants of interest included age, gender, marital status, urban versus rural residence, place of birth and cause of death. We were particularly interested in associations with death in extended care facilities and at home.

## Methods

End of life care in British Columbia is delivered in different locations, by different teams, with relatively good integration in the delivery of patient care. At the provincial level, all patients with less than 6 months life expectancy are enrolled in the British Columbia Palliative Care Benefits Program which entitles them to receive medications, equipment and home support. In the tertiary care hospitals there are Palliative Care Units, and a Palliative Care consult team for patients in other parts of hospital with palliative care needs. There are free-standing hospices across the province for more stable patients. The home-hospice program is integrated with the community home care program and the latter provides home and nursing support to patients at home. Clinical nurse specialists in palliative care and palliative care physicians provide support to the primary care physicians and nurses in the community. There is also a 24 hour toll free phone line, staffed by palliative care specialists, for physicians in remote areas with no direct access to palliative care specialists.

Our study was restricted to vital statistics data and we studied all adult deaths (≥19 years of age) in British Columbia for the years 2004 to 2008. Information about these deaths was obtained from the Vital Statistics Agency of British Columbia, which compiled this data from death registrations. The single underlying cause of death, coded using the International Classification of Diseases 10th revision (ICD-10), was based on the hierarchical cause of death information in the death registration [6,7].

Our study design and analysis used the proportionate mortality method to study associations between the determinants of interest and place of death. This study design is best conceptualized as a population-based case control study, with instances of a particular type of death (e.g., home death) treated as a case and an instance of another type of death serving as a control [8,9]. Cases were defined by the specific place of death and separate analyses were carried out to determine the determinants of home death, death in extended care and hospital death. The determinants of interest included age at death (<50, 50–59, 60–69,70–79, 80–89 and ≥90 years), sex (male and female), marital status (married, never married, widowed and other, with the latter including separated, divorced and common law status), residence status (Metro Vancouver, Victoria, other urban and rural residence), place of birth (Canada, Europe, USA, China, India, Hong Kong, Philippines and other) and the underlying cause of death. The distinction between residence in an urban versus a rural area was made using postal codes [10]. The name of the city or town where death occurred was used to further categorize urban locations into Vancouver, Victoria and other urban areas.

The single underlying cause of death coded in the death registration was categorized into one of 6 leading causes of death [3] and included malignant neoplasms (ICD-10 codes C00-C97), cardiovascular disease (I00–I51), cerebrovascular disease (I60–I69), chronic obstructive pulmonary disease (COPD, J40–J44), pneumonia/influenza (J09–J181, J188, J189), vascular/senile dementia (F01-F03, G30-G32) and other causes.

The relation between each determinant and the place of death was examined and rates of death at home, hospital and extended care were calculated for each category of each determinant. Relationships between determinants and place of death were expressed using odds ratios with 95% confidence intervals (incidence density ratios technically, given the case control study design [8,9]). P values were used to guide inference and a two-sides P value of <0.05 was considered statistically significant. Logistic regression was used to estimate the independent effects of each determinant with the final model including variates based on all determinants of interest. Ethics approval was obtained from the University of British Columbia Clinical Research Ethics Board.

## Results

There were 153,111 adult deaths in British Columbia between 2004 and 2008. Of these, 25,241 (16.5%) occurred at home, 44,349 (29.0%) occurred in extended care facilities, 78,161 (51.0%) occurred in hospital and 5,360 (3.5%) occurred elsewhere. Table 1 shows the characteristics of the decedents by place of death. The proportion of home deaths decreased with age, while the proportion of deaths in extended care facilities was highest among deaths ≥90 years. The proportions of deaths in hospital were lowest at the extremes of age. The proportion of female deaths that occurred at home was lower than the same proportion among males, while the reverse was true for deaths in extended care facilities. Among deaths of people in rural areas, home deaths were more frequent, while deaths in extended care were less frequent compared with deaths in urban locations. Similarly, place of death differed by marital status, place of birth and cause of death (Table 1).

**Table 1:** Characteristics of decedents by place of death, British Columbia, Canada, 2004 to 2008

**Characteristic**	**Home**		**Extended care**		**Hospital**		**Other**		**Total**
	**No.**	**%**	**No.**	**%**	**No.**	**%**	**No.**	**%**	
Age (years) <50	3,181	28.7	761	6.9	4,498	40.6	2,639	23.8	11,079
50–59	3,681	28.6	1,596	12.4	6,620	51.5	955	7.4	12,852
60–69	4,561	23.8	2,986	15.6	10,847	56.7	739	3.9	19,133
70–79	6,040	17.7	7,576	22.3	19,868	58.4	556	1.6	34,040
80–89	5,919	11.8	18,155	35.8	26,331	51.8	384	0.8	50,789
≥90	1,859	7.4	13,275	52.6	9,997	39.6	87	0.3	25,218
Sex – Female	10,226	13.7	26,865	36.0	36,249	48.6	1,238	1.7	74,578
Male	15,015	19.1	17,484	22.3	41,912	53.4	4,122	5.3	78,533
Marital status - Married	10,865	17.5	13,527	21.8	35,949	57.8	1,824	2.9	62,165
Never married	3,574	24.2	3,057	20.7	6,357	43.1	1,777	12.0	14,765
Widowed	6,116	11.0	23,144	41.7	25,753	46.4	495	0.9	55,508
Other	4,686	22.7	4,621	22.4	10,102	48.9	1,264	6.1	20,673
Residence - Vancouver	8,813	14.6	16,503	27.3	33,817	55.8	1,430	2.4	60,563
Victoria	2,513	18.4	5,381	38.9	5,621	40.7	287	2.1	13,563
Other city	9,966	16.8	18,410	31.1	28,437	48.0	2,480	4.2	59,293
Rural	3,924	20.2	4,055	20.9	10,286	52.9	1,163	6.0	19,428
Birth country - Canada	17,399	17.0	29,445	28.8	51,357	50.3	3,988	3.9	102,189
Europe	4,941	15.7	10,424	33.1	15,564	49.4	606	1.9	31,535
USA	646	16.7	1,230	31.8	1,810	46.8	178	4.6	3,864
China	459	9.4	1,260	25.9	3,067	63.1	75	1.5	4,861
Hong Kong	97	13.5	170	23.7	428	59.7	22	3.1	717
India	458	16.2	314	11.1	1,917	67.6	146	5.2	2,835
Philippines	133	15.5	156	18.2	542	63.2	27	3.2	858
Other	1,108	17.7	1,350	21.6	3,476	55.6	318	5.1	6,252
Cause of death - Cancer	8,034	18.7	12,197	28.3	22,530	52.4	271	0.6	43,032
Cardiovascular	7,324	21.1	9,022	26.1	17,411	50.1	984	2.8	34,741
Cerebrovascular	633	5.6	4,174	36.7	6,507	57.3	51	0.5	11,365
COPD	820	12.4	1,779	27.0	3,950	60.0	40	0.6	6,589
Pneumonia/Influenza	244	5.6	1,665	38.0	2,439	55.7	33	0.8	4,381
Dementia	229	2.6	6,532	75.2	1,910	22.0	12	0.1	8,683
Other	7,957	13.0	8,980	20.3	23,414	52.8	3,969	9.0	44,320
Year of death – 2004	5,070	17.1	7,988	26.9	15,554	52.4	1,051	3.5	29,633
2005	5,065	16.8	8,258	27.5	15,639	52.0	1,107	3.7	30,069
2006	5,029	16.5	9,176	30.1	15,263	50.1	1,023	3.4	30,491
2007	5,043	16.2	9,709	31.3	15,236	49.1	1,069	3.4	31,057
2008	5,034	15.8	9,218	29.0	16,469	51.7	1,110	3.5	31,831

Table 2 shows the crude and adjusted odds ratios between person characteristics and home deaths. Deaths of people aged <50, 50–59 and 60–69 years were more likely to occur at home than deaths of people at 70–79 years of age. Conversely, deaths of people aged 80–89 and ≥90 years were less likely to occur at home than deaths of people 70–79 years. Male deaths were more likely to occur at home than female deaths. Deaths among never married people were more likely to occur at home compared with deaths among the married. Comparisons by residence status showed that deaths in Victoria, other urban areas and rural areas were more likely to occur at home than deaths in Vancouver. Deaths from cardiovascular disease were more likely to occur at home compared with deaths from cancer, whereas deaths from cerebrovascular disease, Chronic Obstructive Pulmonary Disease (COPD), pneumonia/influenza, dementia and other causes were less likely to occur at home than cancer deaths. Compared with deaths in 2004, deaths in subsequent years were less likely to occur at home.

**Table 2:** Crude and adjusted associations between decedent characteristics and home death, British Columbia, Canada, 2004 to 2008

**Characteristic**	**Crude odds ratio**	**95% CI**	Adjusted odds ratio*	**95% CI**	**P value**
Age (years) <50	1.87	1.78–1.96	1.74	1.64–1.84	<0.0001
50–59	1.86	1.78–1.95	1.75	1.66–1.83	<0.0001
60–69	1.45	1.39–1.52	1.38	1.32–1.44	<0.0001
70–79	1.00	–	1.00	–	–
80–89	0.61	0.59–0.64	0.66	0.63–0.68	<0.0001
≥90	0.37	0.35–0.39	0.42	0.39–0.44	<0.0001
Sex – Female	1.00	–	1.00	–	–
Male	1.49	1.45–1.53	1.16	1.12–1.19	<0.0001
Marital status – Married	1.00	–	1.00	–	–
Never married	1.51	1.44–1.57	1.21	1.15–1.27	<0.0001
Widowed	0.59	0.57–0.61	0.97	0.93–1.01	0.12
Other	1.38	1.33–1.44	1.18	1.13–1.23	<0.0001
Residence – Vancouver	1.00	–	1.00	–	–
Victoria	1.32	1.26–1.39	1.43	1.36–1.51	<0.0001
Other city	1.19	1.15–1.22	1.17	1.13–1.21	<0.0001
Rural	1.49	1.43–1.56	1.32	1.26–1.38	<0.0001
Cause of death – Cancer	1.00	–	1.00	–	–
Cardiovascular	1.16	1.12–1.21	1.56	1.51–1.62	<0.0001
Cerebrovascular	0.26	0.24–0,28	0.36	0.33–0.39	<0.0001
COPD	0.62	0.57–0.67	0.77	0.72–0.84	<0.0001
Pneumonia/Influenza	0.26	0.23–0.29	0.38	0.34–0.44	<0.0001
Dementia	0.12	0.10–0.14	0.20	0.18–0.23	<0.0001
Other	0.95	0.92–0.99	0.93	0.90–0.97	0.0002
Year of death – 2004	1.00	–	1.00	–	–
2005	0.98	0.94–1.03	0.98	0.94–1.03	0.43
2006	0.96	0.92–1.00	0.98	0.94–1.02	0.32
2007	0.94	0.90–0.98	0.96	0.92–1.00	0.06
2008	0.91	0.87–0.95	0.93	0.89–0.97	0.002

* Adjusted odds ratios obtained from logistic models that also controlled for place of birth.

The crude and adjusted associations between person characteristics and deaths in extended care are shown in Table 3. Deaths among older people were more likely to occur in extended care, while males were less likely to die in extended care as compared with females. Analysis by marital status showed that deaths of married people were less likely to occur in extended care facilities in comparison with deaths of those never married or widowed. Deaths in Victoria and other urban areas were more likely to occur in extended care compared with deaths in Metro Vancouver, while deaths in rural areas were less likely to occur in extended care. Analysis by cause of death revealed that deaths from cardiovascular disease, cerebrovascular disease, COPD, pneumonia/influenza, and other causes were less likely to occur in extended care facilities as compared with cancer deaths but deaths due to dementia were much more likely to occur in extended care facilities. Deaths in extended care increased in frequency after 2004 (Table 3).

**Table 3:** Crude and adjusted associations between decedent characteristics and death in an extended care facility, British Columbia, Canada, 2004 to 2008

**Characteristic**	**Crude odds ratio**	**95% CI**	Adjusted odds ratio*	**95% CI**	**P value**
Age (years) <50	0.26	0.24–0.28	0.29	0.26–0.31	<0.0001
50–59	0.50	0.47–0.53	0.52	0.49–0.55	<0.0001
60–69	0.65	0.62–0.68	0.66	0.63–0.69	<0.0001
70–79	1.00	–	1.00	–	–
80–89	1.94	1.89–2.01	1.75	1.70–1.81	<0.0001
≥90	3.88	3.75–4.02	3.31	3.19–3.45	<0.0001
Sex – Female	1.00	–	1.00	–	–
Male	0.51	0.50–0.52	0.73	0.71–0.75	<0.0001
Marital status – Married	1.00	–	1.00	–	–
Never married	0.94	0.90–0.98	1.58	1.50–1.66	<0.0001
Widowed	2.57	2.50–2.64	1.42	1.38–1.47	<0.0001
Other	1.04	1–1.08	1.35	1.30–1.41	<0.0001
Residence – Vancouver	1.00	–	1.00	–	–
Victoria	1.70	1.64–1.77	1.59	1.53–1.66	<0.0001
Other city	1.20	1.17–1.23	1.22	1.18–1.26	<0.0001
Rural	0.71	0.68–0.73	0.80	0.76–0.83	<0.0001
Cause of death – Cancer	1.00	–	1.00	–	–
Cardiovascular	0.89	0.86–0.92	0.53	0.51–0.54	<0.0001
Cerebrovascular	1.47	1.41–1.53	0.85	0.81–0.89	<0.0001
COPD	0.94	0.88–0.99	0.62	0.59–0.66	<0.0001
Pneumonia/Influenza	1.55	1.45–1.65	0.80	0.75–0.86	<0.0001
Dementia	7.68	7.28–8.10	3.91	3.69–4.13	<0.0001
Other	0.64	0.62–0.66	0.54	0.52–0.56	<0.0001
Year of death – 2004	1.00	–	1.00	–	–
2005	1.03	0.99–1.07	1.03	0.99–1.07	0.14
2006	1.17	1.13–1.21	1.14	1.10–1.18	<0.0001
2007	1.23	1.19–1.28	1.22	1.17–1.26	<0.0001
2008	1.11	1.07–1.15	1.04	1.00–1.08	0.06

* Adjusted odds ratios obtained from logistic models that also controlled for place of birth.

Table 4 provides the crude and adjusted associations between person characteristics and deaths in hospital. Deaths at the extremes of age were less likely to occur in hospital, while male deaths and deaths of married people were more likely to occur in hospital. Deaths outside Metro Vancouver were less likely to occur in hospital, whereas deaths due to cardiovascular disease, cerebrovascular disease, COPD, pneumonia/influenza, and other causes were more likely to occur in hospital as compared with cancer deaths. On the other hand, deaths from dementia were much less likely to occur in hospital compared with deaths from cancer. There was no significant change in the likelihood of death in hospital between 2004 and 2008, though there were fewer deaths in hospital in 2006 and 2007 as compared with 2004.

**Table 4:** Crude and adjusted associations between decedent characteristics and death in hospital, British Columbia, Canada, 2004 to 2008

**Characteristic**	**Crude odds ratio**	**95% CI**	Adjusted odds ratio*	**95% CI**	**P value**
Age (years) <50	0.49	0.47–0.51	0.51	0.48–0.53	<0.0001
50–59	0.76	0.73–0.79	0.75	0.72–0.79	<0.0001
60–69	0.93	0.90–0.97	0.93	0.89–0.96	<0.0001
70–79	1.00	–	1.00	–	–
80–89	0.77	0.75–0.79	0.85	0.82–0.87	<0.0001
≥90	0.47	0.45–0.48	0.55	0.53–0.58	<0.0001
Sex – Female	1.00	–	1.00	–	–
Male	1.21	1.18–1.24	1.05	1.03–1.07	<0.0001
Marital status – Married	1.00	–	1.00	–	–
Never married	0.55	0.53–0.57	0.60	0.58–0.63	<0.0001
Widowed	0.63	0.62–0.65	0.73	0.71–0.75	<0.0001
Other	0.70	0.68–0.72	0.71	0.68–0.73	<0.0001
Residence – Vancouver	1.00	–	1.00	–	–
Victoria	0.54	0.52–0.56	0.56	0.54–0.58	<0.0001
Other city	0.73	0.72–0.75	0.73	0.71–0.74	<0.0001
Rural	0.89	0.86–0.92	0.86	0.83–0.89	<0.0001
Cause of death – Cancer	1.00	–	1.00	–	–
Cardiovascular	0.91	0.89–0.94	1.05	1.02–1.08	0.004
Cerebrovascular	1.22	1.17–1.27	1.40	1.34–1.46	<0.0001
COPD	1.36	1.29–1.44	1.49	1.41–1.57	<0.0001
Pneumonia/Influenza	1.14	1.07–1.22	1.43	1.34–1.52	<0.0001
Dementia	0.26	0.24–0.27	0.31	0.29–0.33	<0.0001
Other	1.02	0.99–1.05	1.23	1.19–1.26	<0.0001
Year of death – 2004	1.00	–	1.00	–	–
2005	0.98	0.95–1.02	0.99	0.96–1.02	0.58
2006	0.91	0.88–0.94	0.93	0.90–0.96	<0.0001
2007	0.87	0.85–0.90	0.89	0.86–0.92	<0.0001
2008	0.97	0.94–1.00	1.01	0.98–1.04	0.61

* Adjusted odds ratios obtained from logistic models that also controlled for place of birth.

Table 5 shows the association between place of birth and place of death, with deaths of those born in Canada constituting the reference category. Deaths of people born in China and Hong Kong were less likely to occur at home, while deaths of those born in Europe and the USA were more likely to occur at home. Deaths of people born in India, China and the Philippines were significantly less likely to occur in extended care facilities. Finally, people born in China, India and the Philippines were more likely to die in hospital, while those born in Europe and the United States were less likely die in hospital as compared with people born in Canada.

**Table 5:** Crude and adjusted odds ratios expressing the association between place of birth and place of death in British Columbia, 2004 to 2008

**Place of death/**	**Crude odds**	**95% CI**	**Adjusted**	**95% CI**	**P value**
**Place of birth**	**ratio**		odds ratio*		
Place of death: Home					
Place of birth – Canada	1.00	–	1.00	–	–
Europe	0.91	0.88–0.94	1.13	1.09–1.17	<0.0001
USA	0.98	0.90–1.07	1.12	1.03–1.23	0.009
China	0.51	0.46–0.56	0.70	0.63–0.78	<0.0001
Hong Kong	0.76	0.62–0.95	0.73	0.59–0.91	0.004
India	0.94	0.85–1.04	0.97	0.87–1.08	0.56
Philippines	0.89	0.74–1.08	0.92	0.76–1.11	0.43
Other	1.05	0.98–1.12	1.00	0.93–1.07	0.94
Place of death: Extended care facility					
Place of birth – Canada	1.00	–	1.00	–	–
Europe	1.22	1.19–1.25	0.98	0.95–1.01	0.25
USA	1.15	1.08–1.24	0.95	0.88–1.02	0.19
China	0.86	0.81–0.92	0.79	0.74–0.85	<0.0001
Hong Kong	0.77	0.65–0.91	1.09	0.90–1.31	0.39
India	0.31	0.27–0.35	0.35	0.31–0.40	<0.0001
Philippines	0.55	0.46–0.65	0.70	0.58–0.84	<0.0002
Other	0.68	0.64–0.72	0.85	0.80–0.91	<0.0001
Place of death: Hospital					
Place of birth – Canada	1.00	–	1.00	–	–
Europe	0.97	0.94–0.99	0.93	0.91–0.96	<0.0001
USA	0.87	0.82–0.93	0.91	0.85–0.97	0.003
China	1.69	1.59–1.80	1.31	1.23–1.39	<0.0001
Hong Kong	1.47	1.26–1.70	1.14	0.98–1.33	0.09
India	2.07	1.91–2.24	1.69	1.55–1.83	<0.0001
Philippines	1.70	1.48–1.95	1.35	1.17–1.56	<0.0001
Other	1.24	1.18–1.31	1.07	1.02–1.13	0.01

* Adjusted odds ratios obtained from logistic models which controlled for age, sex, marital status, residence, cause of death and year of death.

## Discussion

Our study showed that person characteristics such as age, gender, marital status, residence, cause of death and place of birth were significantly associated with place of death. For instance, age was a strong determinant of place of death, with younger people more likely to die at home and older people more likely to die in extended care. Males were less likely to die in extended care as compared with females and more likely to die at home or in hospital. Perhaps the most intriguing finding of our study was the association between place of birth and place of death; people born in Europe and the United states were more likely to die at home compared with those born in Canada, whereas those born in Asia were less likely to die at home and more likely to die in hospital. People born in China, India and the Philippines were less likely to die in extended care compared with those born in Canada.

The increased likelihood of males dying at home or in hospital has been described previously [5,11], although this finding has not been consistently observed in all settings [12]. Studies have shown that even among residents of nursing homes, males are more likely to die in hospital [13]. Our study shows that adjustment for potential confounders substantially attenuated the association between male gender and hospital death (crude odds ratio 1.21, 95% CI 1.18–1.24 versus adjusted odds ratio 1.05, 95% CI 1.03–1.07). It is likely that the adjusted association is a product of residual confounding because of our use of broad age categories and potential misclassification of other factors. However, the association between male gender and increased home death and decreased death in extended care was stronger and likely represents a sociologic phenomenon.

Studies on cancer deaths show that such deaths are more likely to occur in hospital in both the big urban centres and in rural areas [14-16]. Although in our study 52.4% of cancer deaths occurred in hospital we noted that, relative to other causes of death (except deaths due to dementia), cancer patients were less likely to die in hospital.

In our study, deaths in Metro Vancouver were more likely to occur in hospital compared with deaths in Victoria and other urban areas, while deaths in rural areas were more likely to occur at home and less likely to occur in extended care or in hospital. These findings suggest that rural–urban differences may be due to a lesser degree of social support in urban centers [17,18]. Sparse research limits our understanding of these issues [19], although one possibility that cannot be excluded is the higher concentration of resource intense health services in urban areas and the consequent medicalization of death [20]. Access to home care services may also have an impact on location of death [21].

One of the most interesting findings of our study was the relationship between country of birth and place of death. The increased tendency of immigrants and, specifically, foreign born Asians to die in hospital has been noted in previous studies [1,2,22-28]. In our study, differences were observed in place of death preference among people with place of birth in China, India and the Philippines. These three population subgroups were more likely to die in hospital than people born in Canada. Whereas those born in China were less likely to die at home and extended care and more likely to die in hospital, people born in India and the Philippines were as likely as those born in Canada to die at home though not in extended care facilities. There is little information in the literature on the influence of place of birth and/or ethnicity on place of death. A study from California [23] showed that death in hospital was most common for Asian and Hispanic immigrants and least common for non-Hispanic whites and US-born Asians. Similarly, a study done on cancer patients in the United Kingdom [25] reported that Indian, Pakistani, Bangladeshi and Chinese patients were significantly more likely to die in hospital than White patients. A systematic review on minority ethnic groups and end of life care done in the United Kingdom [28] also showed that Asian patients with cancer were half as likely as non-Asians to die in a hospice, and that elderly Chinese expressed a preference for hospital care. The authors of these different studies have speculated that observed differences were due to cultural preferences, with reluctance among some immigrant subpopulations to utilize hospice services and complete advance directives.

Estimates such as those in our study could be used to inform public health policy and future resource allocation based on anticipated changes to population structure. This is a critical issue as the ageing of the Canadian population will increase the number of deaths per year substantially, with deaths in British Columbia likely to increase by 27% from 2004–08 levels by 2020 and by 50% by 2035 (assuming anticipated changes in population structure [29] and no change in age-specific death rates). Planning for health service delivery needs to account for such changes if resource allocation is to be appropriately directed at end of life services in the community, in extended care facilities and in hospital. For instance, anticipated increases in the absolute number and proportion of elderly decedents, implies a greater need for extended care facilities, given the place of death patterns observed in our study. Similarly, with immigration expected to account for 77.4% of increase in population by 2036 [29], place of birth considerations may be helpful in terms of anticipating demand for end of life services in specific locales.

Our study was based on data abstracted from death registrations which may have some inaccuracies related to the location of death, marital status and single coded underlying cause of death. The usage of postal codes to delineate urban and rural areas represents a standard method of classifying residence status but may not be completely accurate. Place of birth is not an exact determinant of ethnicity but is a pragmatic alternative that is both relevant and informative in this context. Finally, our study was descriptive and focused on where people died and not what kind of services they received as we did not have access to data about end of life services. Location of death should not be used as proxy for the extent of community-based resources as the final location of death may not necessarily be the location of care during most of the end of life [30].

## Conclusions

In summary, our study shows that age, sex, marital status, rural versus urban residence, cause of death and place of birth are significant determinants of death at home, in extended care facility or in the hospital. The findings of our study can be used for the rational and knowledge-based allocation of health care resources including those required for hospital, extended care and community services at the end of life.

## Abbreviations

CI: Confidence Intervals; Vs: Versus; ICD: International Classification of Diseases; USA: United States of America; COPD: Chronic Obstructive Pulmonary Disease; BC: British Columbia.

## Competing interests

The authors declared that they have no competing interest.

## Pre-publication history

The pre-publication history for this paper can be accessed here:

http://www.biomedcentral.com/1472-684X/12/19/prepub
